# Co-constructing collaboration: An evidence-based approach to advance and evaluate equitable global public health research partnerships

**DOI:** 10.1371/journal.pgph.0002481

**Published:** 2023-10-23

**Authors:** Vanessa Amos, Virginia LeBaron, Tuyet Chuong, Catherine E. Elmore, Pawan Kumar Hamal, Bishnu D. Paudel, Amber Steen, Sandhya Chapagain

**Affiliations:** 1 University of Virginia School of Nursing, Charlottesville, Virginia, United States of America; 2 University of Utah College of Nursing, Salt Lake City, Utah, United States of America; 3 National Academy of Medical Sciences, National Trauma Center, Kathmandu, Nepal; 4 National Academy of Medical Sciences (NAMS) Bir Hospital, Kathmandu, Nepal; 5 University of Virginia, Center for Global Health Equity, Charlottesville, Virginia, United States of America; University of the Witwatersrand, SOUTH AFRICA

## Abstract

Equitable global health partnerships are essential to promote innovative research and strengthen research capacity to address critical public health challenges, but how to optimally evaluate such collaborations is unclear. This was a sequential, multi-method study that utilized an electronic survey informed by the literature followed by semi-structured interviews to comprehensively evaluate the experience of participating in a global research-capacity building collaboration between Nepal and U.S. clinicians and investigators. De-identified quantitative survey were analyzed to calculate descriptive and summary statistics, along with crosstabs of each variable by group. Groups were defined based on country-of-origin and Chi Square statistics calculated to assess for statistically significant differences (p<0.05) between groups. Interviews were analyzed using a descriptive qualitative approach to develop an overall thematic map. 22 survey responses (52.4% response rate) were analyzed; 13 (59.1%) from Nepal, 9 (40.9%) from the U.S. Eight participants (4 Nepal; 4 U.S.) were interviewed. Over the course of the project, all participants reported gaining experience and confidence with research. The majority of participants “strongly agreed” there was a shared understanding of goals, priorities and strategies (Nepal, 58.3%, n = 7; U.S., 88.9%, n = 8;) and that power was shared equally (Nepal, 58.3%, n = 7; U.S., 55.6%, n = 5). The over-arching theme that emerged from the interviews was the importance of ‘establishing community’ which participants discussed within the broader context of COVID-19. Overall, team members reported strong bi-directional benefit and a greater emphasis on perceived benefits versus challenges. Our survey tool and interview guide, designed to holistically evaluate the impact of a global partnership across various levels of the Social Ecological Model, with particular attention to power dynamics and equity, can be adapted and used by others engaged in similar research capacity collaborations.

## Introduction

Equitable advancement of public health requires researchers to collaborate and work together across cultures and countries to generate knowledge that is relevant and contextually congruent. This lesson applies to the urgent global health needs brought into stark relief by the COVID-19 pandemic, but it also applies to the pre-pandemic and on-going need for research capacity building in low and middle-income countries (LMICs) in the critical public health areas of mobile health [1–4], management of non-communicable diseases [5, 6], and workforce development and training [7–9]. The importance of developing equitable global health partnerships to promote innovative research and strengthen research capacity in these areas has been widely acknowledged by organizations such as the United Nations Sustainable Development Goals [10], the World Health Organization [11], the National Institutes of Health [12, 13] and the International Council of Nurses [14].

Significant efforts have been undertaken to foster global health partnerships that are co-constructed, equalize power imbalances, and empower those whose voices are too often ignored or dismissed [15–19]. While this is an essential end-goal, developing research partnerships between LMICs and higher income countries (HICs), (also referred to as North-South partnerships), can be challenging [20, 21]. Funding constraints, infrastructure limitations, normative sociocultural and communication differences, economic disparities, competing personal and institutional priorities, conflicts due to organizational dynamics and individual personalities, and natural and geopolitical crises can all converge to make developing–and sustaining–equitable global health partnerships additionally complex. Importantly, with the formation of global partnerships comes an additional challenge of knowing how to best evaluate their impact and effectiveness [22].

This paper presents findings from our post-project evaluation of a global partnership between clinicians and researchers in Nepal and the U.S. The collaboration builds upon long-standing relationships forged between oncology and palliative care clinicians and researchers in the U.S. (based primarily at the University of Virginia, or UVA) and in Nepal (associated with the non-profit palliative care advocacy organization, the Nepalese Association of Palliative Care, or NAPCare), that first began in 2004. This paper presents results from the evaluation of a 3-year global collaboration, supported by the National Institutes of Health (NIH) Fogarty International Center (April 2018 –April 2021) that occurred during the COVID-19 global pandemic. The parent study aimed to develop a mobile application (‘app’) to support Nepali healthcare providers (HCPs) in managing cancer pain [23]. Another key deliverable from the parent grant was the creation of a ‘Virtual Library’–a web-based repository of research-related resources designed to support clinical investigators in resource-constrained settings [24]. Results related to the design and pilot testing of the mobile app [25, 26] and creation of the Virtual Library [27] have been previously reported.

The purpose of this study was to evaluate the experience of participating in the Nepal-U.S. research collaboration to inform next steps of this project and offer an evidence-informed approach to holistically understand the impact of global health partnerships that may be helpful to others engaged in similar work. This research contributes to the existing global research capacity literature by evaluating this partnership using both quantitative and qualitative approaches to fully understand the perspective of both HIC and LMIC interdisciplinary team members, with particular attention to equity and power dynamics. It further advances the field by offering details regarding the creation and implementation of a novel global collaboration evaluation tool, all within the context of the COVID-19 pandemic.

## Methods

### Ethics statement

Approval for this research was granted by the Nepal Health Research Council and the University of Virginia Social and Behavioral Science Institutional Review Board (IRB), and all participants provided informed written consent prior to data collection. No minors were included in this study. First authors (VA, VL) had access to potentially identifiable data during interview data collection; all identifiers were removed for data analysis and de-identified interview transcripts stored securely in compliance with institutional policies. All team members were invited to participate as co-authors in this manuscript and to help with interpretation of de-identified aggregate and preliminary results. In this way, we aimed to utilize a participatory and collaborative approach with this publication.

### Overview

This sequential, multi-method study utilized an electronic survey followed by semi-structured interviews to comprehensively evaluate the experience of participating in a global research-capacity building collaboration between Nepal and U.S. clinicians and investigators that occurred during the COVID-19 global pandemic. Participants were recruited and enrolled between August–December 2021.

### Surveys

#### Survey development

The primary goal of our survey was to assess research participants’ experiences related to our capacity-building partnership. An important secondary goal was to develop a potential tool to help others interested in comprehensively evaluating cross-global collaborations. We initially aimed to use a pre-existing and validated assessment tool and found many interesting and helpful examples. For example, Holden et al.’s validated research capacity and culture tool from Queensland, Australia had high engagement with its participants [28], as did Boissevain et al.’s UVA-UNIVEN web-based, unvalidated survey used in Limpopo, South Africa [29]. Oetzal et al.’s review of community-based participatory research projects based in the United States was also useful, particularly in highlighting the interplay between the community and researchers [30]. However, the existing evaluation tools we discovered did not capture the full breadth of our vision of research capacity as related to our study aims. We wished to assess trust, equity, and collaboration [29–31], financial and budgetary constraints [32], and, importantly, to explore the impact of the collaboration across all levels of the Social Ecological Model [33] (individual; institutional; country/system) from participants of both LMIC and HIC countries [28]. Huber et al’s review [22] of research capacity monitoring and evaluation tools noted additional limitations we sought to avoid, including an absent, or unclear, definition of ‘research capacity,’ lack of standardized reporting, and a limited focus on organizational research capacity, especially in LMICs. Ultimately, we created a customized survey informed by the existing literature to comprehensively evaluate our research capacity efforts. We propose this survey as part of an “evaluation toolkit” (see S1 Text).

Early-stage conceptualization of our survey began with input from the UVA Center for Survey Research (CSR) and lead investigators from both the U.S. and Nepal teams (n = 6). These discussions confirmed key objectives of the survey, including alignment with the aims of the parent award related to building research capacity for cancer care and assessing the impact of the collaboration across all levels of the Social Ecological Model [33]. We confirmed with Nepali partners that English was the preferred language in which to conduct the survey. We opted to use categorical response options with qualitative labels versus numerical 7- or 10-point Likert scales. This was an intentional survey design choice to enhance clarity and yield more reliable data, based on both feedback from past Nepali survey participants [25] and the existing literature [34, 35].

Our 49-item survey (see S1 Text) was designed using Qualtrics and consisted of four sections: demographics (10 items), research experiences (including the impact of COVID-19 on the project; 13 items), research capacity building (20 items), and open-ended, free-text responses (6 items). Survey items about research capacity building were conceptually informed by a review of the literature [28, 29, 32, 36, 37], and included items related to communication (n = 4), trust (n = 2), decision making (n = 3), sustainability (n = 4), resource availability (n = 2), conflict management (n = 6), finances/budget (n = 2), and motivators and barriers to research (n = 4). Open-ended, free text responses (n = 6) queried personal experiences about participation, including the option to share any additional thoughts or perspectives. For the purposes of this project and survey, we defined research capacity as “as the ability to engage in, perform, or carry out quality research” [38] and this definition was included at the beginning of the survey. The last survey item invited respondents to participate in a follow-up one-on-one interview to further discuss their experiences with the global research collaboration.

Survey pilot testing was conducted with 4 graduate and 3 undergraduate (n = 7) U.S. nursing students not directly affiliated with the project. Survey questions were reviewed for content, clarity, length of time to complete, and presentation on various devices (laptop; desktop; mobile device). After pilot testing, the survey was iteratively revised and finalized by the first authors. Prior to survey deployment, a native Nepali nurse, fluent in both English and Nepali, reviewed all final survey items to ensure clarity and contextual and cultural congruence.

#### Survey deployment

All individuals (except first authors VA and VL, who led survey development), including faculty, clinical partners, students, staff, and consultants, who were involved with the global collaboration in some capacity, administratively or scientifically, at any point during the grant period were invited to participate in the survey via an anonymous email link. All survey respondents provided informed consent prior to data collection by confirming review and agreement with the IRB-approved written consent form. The initial invitation was sent August 2021, approximately 3 months after the parent study ended. It remained open for 2.5 weeks, with reminders sent at one week, two weeks, and two days prior to its closing.

#### Survey data analysis

De-identified quantitative survey responses were exported from Qualtrics to SPSS (v 28.0), cleaned, and verified. Descriptive and summary statistics for all survey items were calculated, along with crosstabs of each variable by group. Groups were defined based on country-of-origin (Nepal vs. U.S.). Chi Square statistics were then calculated to assess for statistically significant differences (p = <0.05) between groups.

Free text survey responses were exported to a Microsoft Word document and organized by survey item (e.g, all responses to a particular question were grouped together; see Table 2). Responses were then reviewed in aggregate and summarized using a basic descriptive content analysis approach. Our goal with this analysis was not to conduct qualitative analysis with a high level of abstraction, but, consistent with a descriptive approach, to remain close to our data and more concretely represent participant responses [39].

### Interviews

#### Interview data collection

To qualitatively evaluate the collaboration, we conducted semi-structured interviews (October–December 2021), with a subset of survey respondents who indicated they would like to be interviewed after completing the survey. All team members also received an emailed invitation approximately one week after the survey closed to capture any further individuals who wanted to participate in the interview phase.

The semi-structured interview guide was also informed by a review of the global research-capacity literature [22, 28–30, 36, 38], and is the second component of our proposed “evaluation toolkit” (see S2 Text). Interview questions were designed to elicit the participants’ overall experiences during the collaboration and, while not directly linked to the quantitative survey results, did seek to provide additional context to those results. A particular goal of the interviews was to explore how participants viewed the relative success or failure of such collaborations and how research capacity was impacted across levels of the Social Ecological Model [33]. Given its temporal relevance, participants were also asked about their perceived impact of the COVID-19 pandemic on the collaboration. Interviews were conducted over Zoom, audio-recorded with permission and averaged ~30 minutes each. To reduce social desirability bias, interviews were conducted by a ‘neutral’ individual (VA) not previously known to participants and not affiliated with the parent study.

#### Interview data analysis

Audio transcripts were de-identified, cleaned, verified, and imported into NVivo (v1.6.23) for analysis. A descriptive, qualitative approach was utilized with the goal to stay close to the data versus achieve a high level of abstraction [39]. Transcripts were coded line-by-line; the units of analysis were short phrases or clusters of words. Transcripts were first analyzed using inductive, open codes, and then deductively using an *a priori* coding schema that mapped onto the interview guide. Inductive coding allowed for data to inform initial analysis and the deductive coding allowed for the exploration of more directed research questions [40]. Codes that emerged from both inductive and deductive analysis were compared and then collapsed into related categories within NVivo. Categories were also confirmed by magnitude coding, a coding method that considers how frequently a given code appears across and within transcripts [40]. Codes and categories were then further explored and verified through a manual ‘sticky note and whiteboard’ exercise (completed by VA and VL) to reach consensus regarding larger themes and categories. This involved writing each code on individual sticky-notes and displaying them on a whiteboard to identify additional patterns or connections among codes that may not have been evident within NVivo. These connections were then graphically represented in a qualitative code map. Preliminary qualitative findings were further shared with interview participants over email (e.g., member-checking [41]) to confirm proposed themes. Five out of the 8 interview participants (62.5%) responded and verified that the proposed themes and figure accurately represented their experience.

## Results

### Survey results

The survey link was emailed to 42 potential respondents. 23 respondents opened the survey; 3 were partially completed, 1 of which was discarded for completing < 50%. In total, 22 surveys (52.4% response rate) were used for analysis. Results are summarized below by survey item categories (statistical significance, when present, is noted).

### Demographics

13 survey respondents (59.1%) were from Nepal, 9 (40.9%) from the U.S. 88.9%, (n = 8) of U.S. team members identified as female. Nepal participants identified as female, 53.8% (n = 7) and male, 46.2% (n = 6). All (100%, n = 13) respondents from Nepal identified as a HCP, most were physicians (69.2%, n = 9). Only two participants from the U.S. team identified as HCPs (22.2%; n = 2); both (100%) were nurses. If identifying as a HCP, the majority of participants (80%, n = 12) had greater than 10-years of experience and provided direct patient care more than 50% of the time. Over half (55.6%; n = 5) of U.S. team members identified as either an undergraduate or graduate student; there were no students from Nepal. Statistically significant differences were found in country-of-origin affiliation (p = 0.011); and between the Nepal and U.S. groups related being a HCP (p<0.001); length of time being a HCP (p = 0.014) and identifying as a student (p = 0.002). See Table 1 for full demographic details.

**Table 1:** Demographics of the survey sample, overall, and by country affiliation.

	Total	Nepal	U.S.
	*n (%)	*n (%)	*n (%)
Sample**	22 (100)	13 (59.1)	9 (40.9)
**Age (years)**			
18–30	5 (22.7)	1 (7.7)	4 (44.4)
31–40	9 (40.9)	7 (53.8)	2 (22.2)
41–50	5 (22.7)	3 (23.1)	2 (22.2)
51–60	2 (9.1)	2 (15.4)	0 (0)
Over 60 years old	1 (4.5)	0 (0)	1 (11.1)
**Gender Identity (self-identified)**			
Female	15 (68.2)	7 (53.8)	8 (88.9)
Male	7 (31.8)	6 (46.2)	1 (11.1)
**Student Role (undergraduate or graduate)** **			
Yes	5 (22.7)	0 (0)	5 (55.6)
No	17 (77.3)	13 (100)	4 (44.4)
**Trained Healthcare Provider** **			
No	7 (31.8)	0 (0)	7 (77.8)
Yes^	15 (68.2)	13 (100)	2 (22.2)
Type of healthcare provider^			
Nurse	6 (27.3)	4 (30.8)	2 (100)
Physician	9 (40.9)	9 (69.2)	0 (0)
Total years working as a healthcare provider**^			
Less than 5	1 (6.7)	0 (0)	1 (50)
6 to 10	2 (13.3)	1 (7.7)	1 (50)
11 to 20	8 (53.3)	8 (61.5)	0 (0)
20 or more	4 (26.7)	4 (30.8)	0 (0)
Percent of time providing direct patient care^			
0–25%	2 (13.3)	1 (7.7)	1 (50)
26–50%	1 (6.7)	1 (7.7)	0 (0)
51–75%	4 (26.7)	4 (30.8)	0 (0)
76–100%	8 (53.3)	7 (53.8)	1 (50)

*Column percentages

**Statistically significant difference between the Nepal and U.S. groups, p < .05

^Questions below only answered by those who answered "yes" to being a healthcare provider (n = 15)

#### Overall experience participating in the collaboration

**Fig 1:**
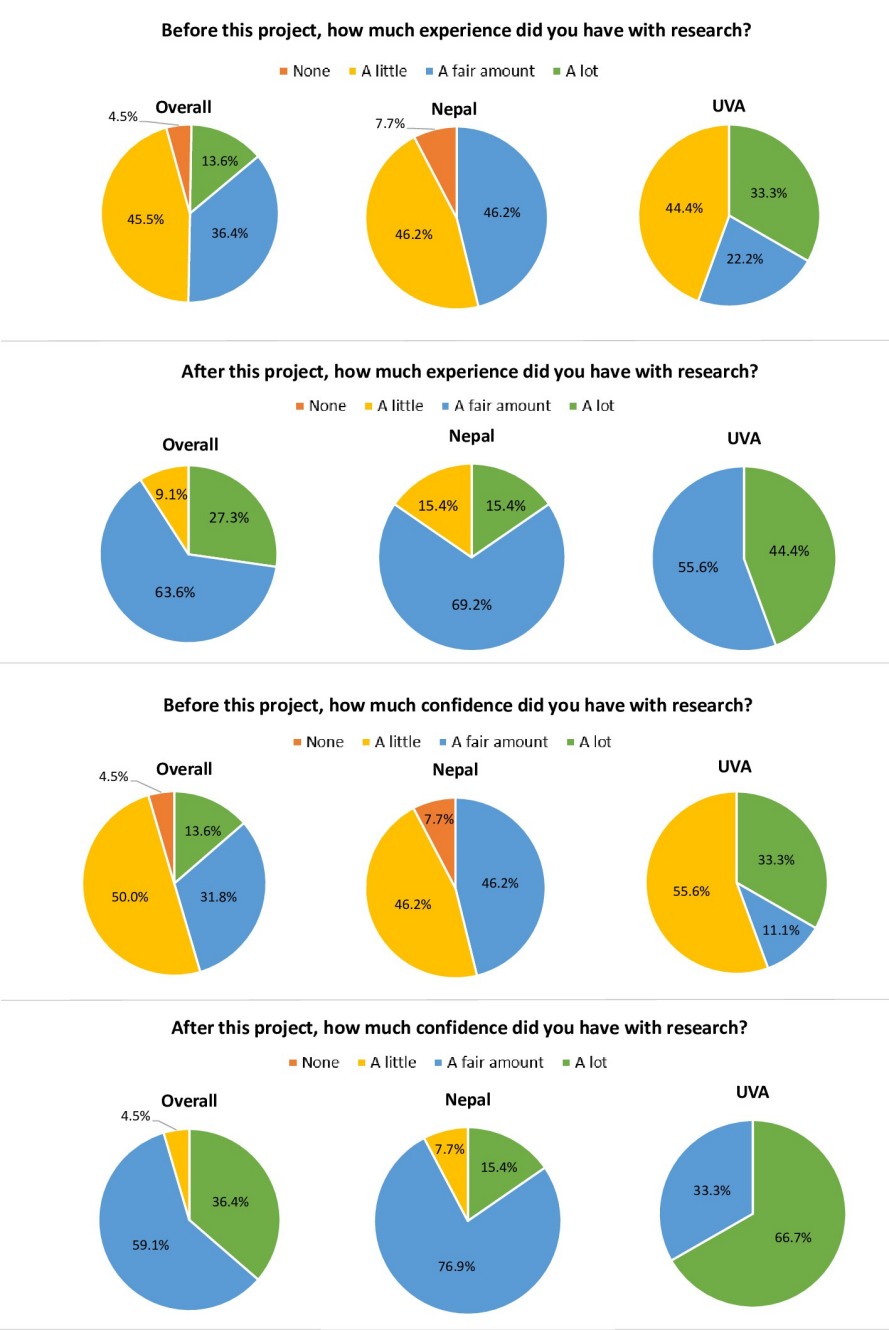
Comparison of pre and post-project experience and confidence related to research, overall, and by group.

For the overall sample, the majority of participants rated their baseline (pre-project) research related experience (45.5%, n = 10) and confidence (50%, n = 11) as “a little” (Fig 1). Over the course of the project, all participants reported gaining experience and confidence with research. Specifically, participants reported a pre- to post-project increase in having “a fair amount” (36.4%, n = 6 to 63.6%, n = 14) or “a lot” (13.6%, n = 3 to 27.3%, n = 6) of research experience. Similarly, respondents reported a pre- to post-project increase in having a “fair amount” (31.8%, n = 7 to 59.1%, n = 13) or “a lot” (13.6%, n = 3 to 36.4%, n = 8) of confidence with research. Changes in self-reported pre- and post-project confidence levels were statistically significant (p = 0.04) between the U.S. and Nepal groups. Additionally, when queried about overall learning with the project, 59.1% (n = 13) of the total sample reported experiencing a “fair amount” of overall learning during the project. 76.9% (n = 10) Nepal participants from Nepal indicated they learned “a fair amount,” while U.S. participants were more likely to report having learned “a lot” during the project (62.5%, n = 5); this difference was statistically significant (p = 0.02). For further context, when participants were asked, “If you were asked to be part of another global research team, what would you say?,” all but one team member (n = 21/22; 95.5%) answered ‘Yes, definitely.’ Specifically, all of the Nepali respondents (n = 13/13; 100%) answered ‘Yes, definitely’; one participant (U.S. team member) responded ‘Maybe I’m not sure,’ (1/22; .04%) and zero (n = 0; 0%) participants responded ‘No, definitely not.”

When asked about motivations for joining the project (a select-all-that-apply item), Nepal team members rated the “opportunity to participate in future similar projects” as their highest motivator (92.3%, n = 12), followed by the chance to “develop and learn skills” (84.6%, n = 11), and then either “career advancement” (76.9%, n = 10) or “publication opportunities” (76.9%, n = 10). All U.S. team members reported their top motivator as the chance to “develop and learn skills” (100%, n = 9), followed by “opportunity to participate in future, similar projects” (88.9%, n = 8), and then “improve patient care” (77.8%, n = 4). The most frequently reported barrier to participation for both groups included other work priorities (Nepal, 61.5%, n = 8; U.S., 33.3%, n = 3). The second highest barrier to participation included COVID-19 for Nepal team members (53.8%, n = 7) or other personal or family commitments for U.S. (22.2%, n = 2).

#### Research capacity impact, sustainability, resources, and role clarity

**Fig 2:**
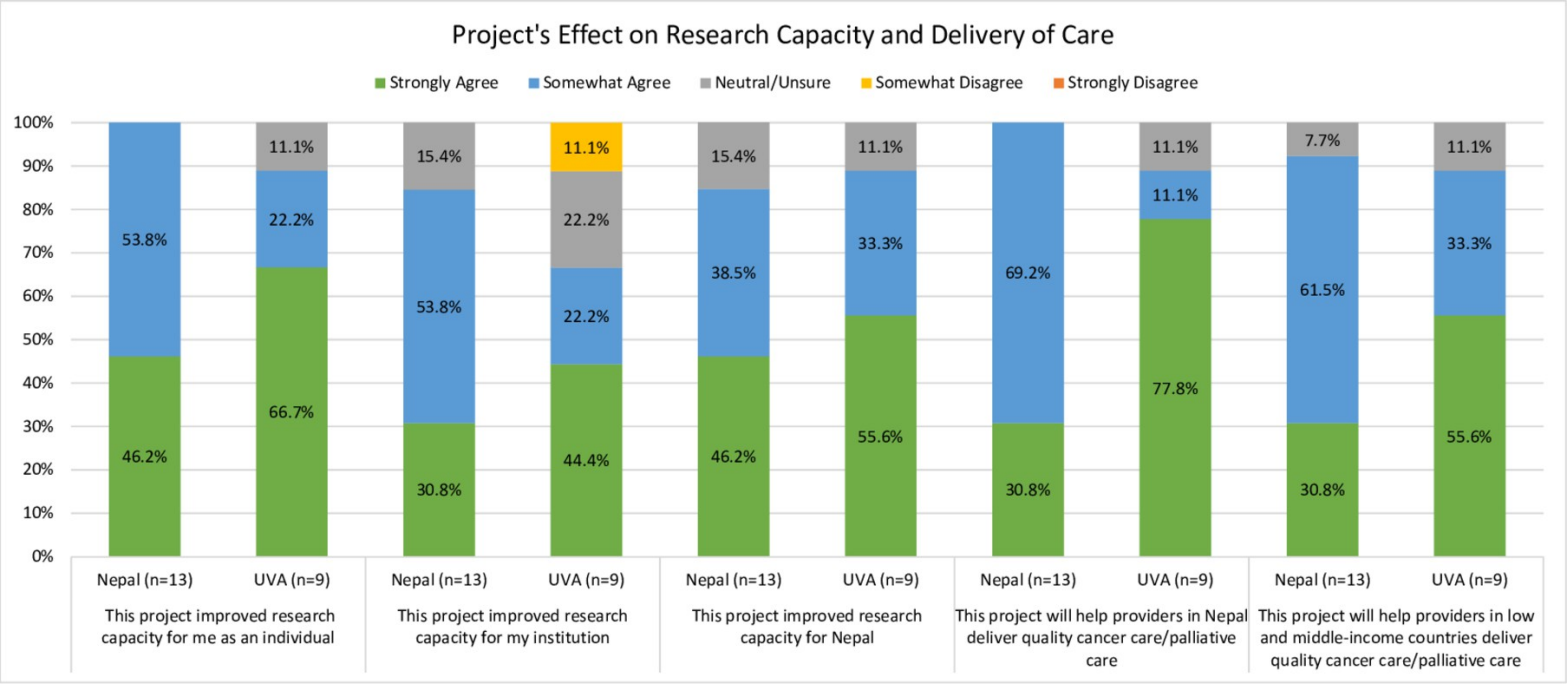
Comparison of project impact on research capacity at the individual, institutional, and country levels, by group.

Survey respondents were asked to reflect upon how the collaboration may have increased research capacity for them as an individual, for their institution, and for Nepal at-large (Fig 2). 46.2% (n = 6) and 66.7% (n = 6) of Nepal and U.S. team members, respectively, “strongly agreed” that the project improved research capacity for them as an individual. Similarly, 46.2% (n = 6) of Nepal team members and 55.6% (n = 5) of U.S. team members “strongly agreed” that the project improved research capacity for the country of Nepal. There were differing perceptions of whether the project improved research capacity for the respondent’s own institution for both Nepal and the U.S.: 30.8% (n = 4) from the Nepal team and 44.4% (n = 4) the U.S. team selected “strongly agree.” U.S. team members (77.8%, n = 7) were also more likely than Nepal team members (30.8%, n = 4) to “strongly agree” the project improved the ability of HCP in Nepal to deliver quality cancer/palliative care, and that the project would help HCP in other (non-Nepal) LMICs deliver quality cancer/palliative care (U.S., 55.6%, n = 4; Nepal, 30.8%, n = 4).

**Fig 3:**
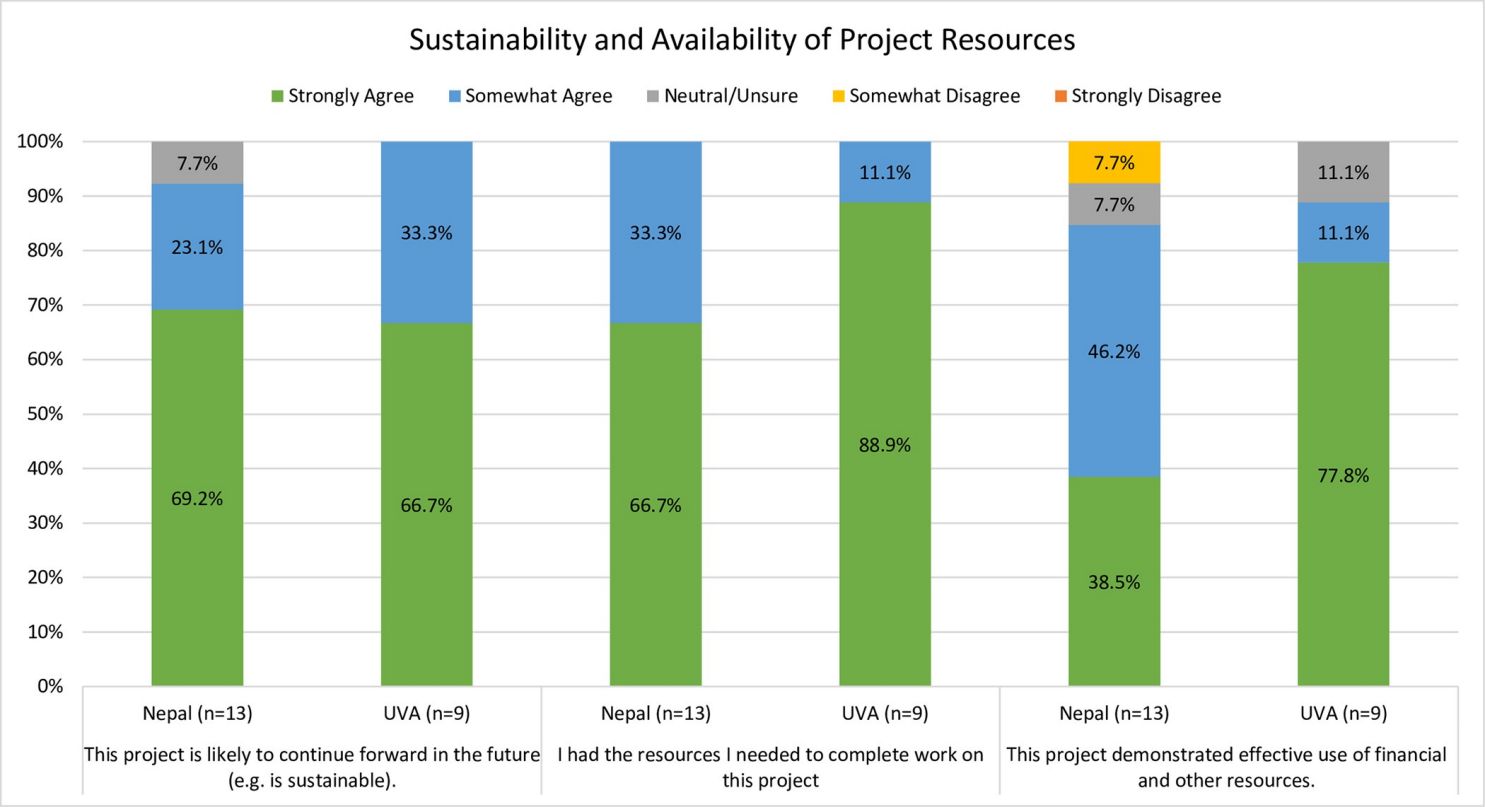
Comparison of project sustainability and resources by group.

When asked about sustainability and resources (Fig 3), the majority from both the U.S. (66.7%, n = 6) and Nepal (69.2%, n = 9) “strongly agreed” the project was “likely to continue forward in the future” and that “I had the resources I needed to complete work on this project” (U.S., 88.9%, n = 8; Nepal, 66.7%, n = 8). A higher percentage of U.S. participants (77.8%, n = 7) indicated they “strongly agreed” there was effective use of financial and other resources, compared to the Nepal team, which more likely to “somewhat agree” (46.2%, n = 6).

**Fig 4:**
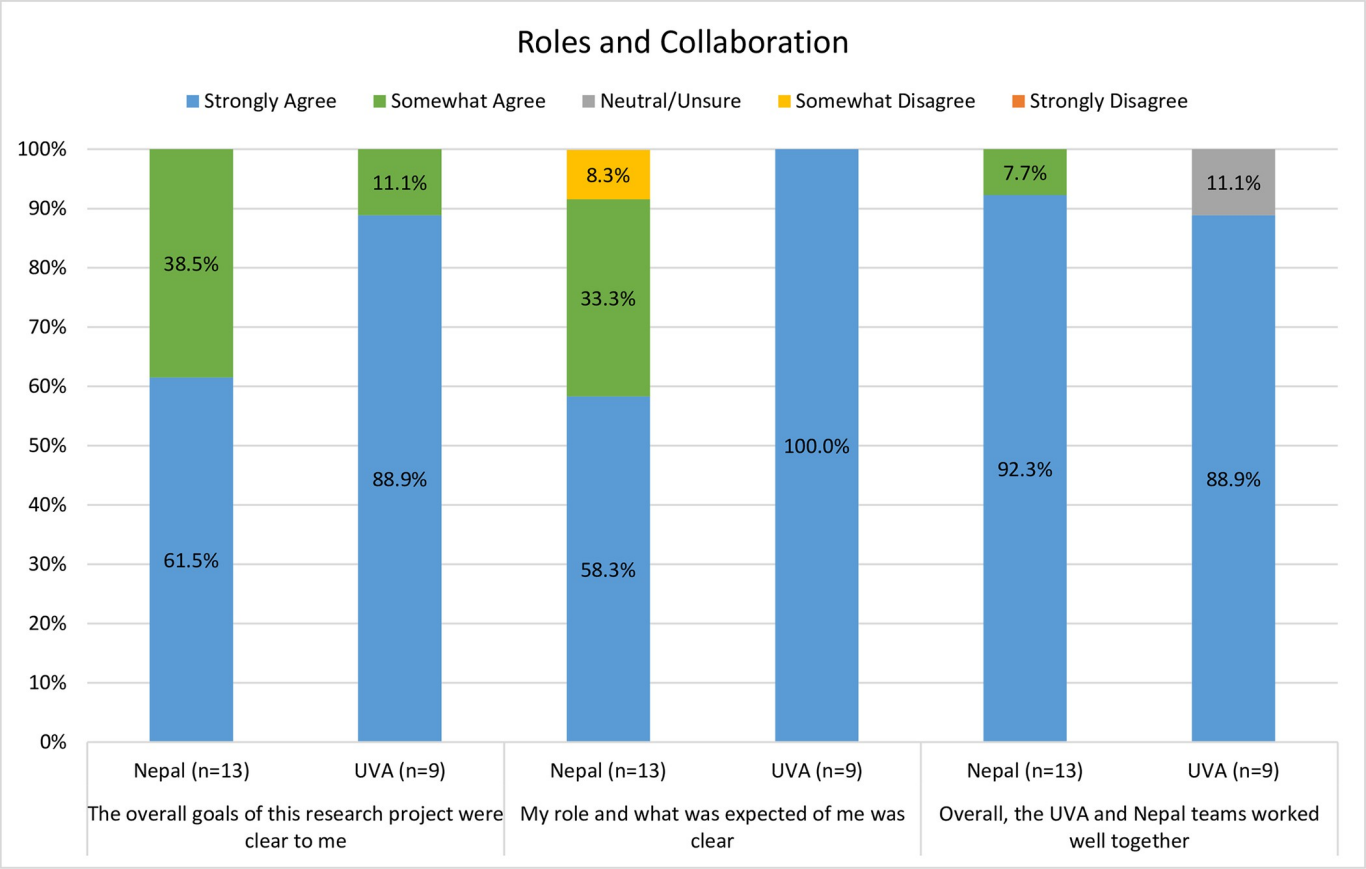
Comparison of role and project clarity and overall collaboration between groups.

61.5% (n = 8) of Nepal team members and 88.9% (n = 8) of U.S. team members “strongly agreed” that the “overall goals of the project were clear.” All U.S. team members (100%, n = 9) and 58.3% (n = 7) of Nepal team members “strongly agreed” that their individual roles were clear. Importantly, the majority of participants “strongly agreed” that the UVA and Nepal groups worked well together (Nepal, 92.3%, n = 12; UVA, 88.9%, n = 8). (Fig 4).

#### Conflict, decision making, mutual benefit, and shared power

**Fig 5:**
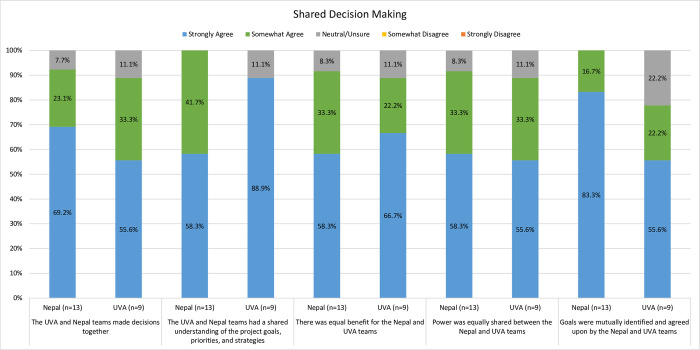
Comparison of perceived shared decision making between groups.

The majority of participants (Nepal, 92.3%, n = 12; U.S., 66.7%, n = 6) reported some conflict during the collaboration, but it was resolved in a way that did not impede progress. Additionally, 100% (n = 9) of U.S. respondents and all but one Nepal respondent (92.3%, n = 12) reported, “Yes, always” when asked if they felt their voices and opinions were heard and respected during the project (the additional Nepal team member responded “Sometimes” to this question, 7.7%, n = 1). Importantly, the majority of participants (Nepal, 58.3%, n = 7; U.S., 88.9%, n = 8;) “strongly agreed” there was a shared understanding of goals, priorities and strategies and that project goals were mutually identified and agreed upon (Nepal, 83.3%, n = 10; U.S., 55.6%, n = 5). More than half of both groups (Nepal, 58.3%, n = 7; U.S., 55.6%, n = 5) “strongly agreed” power was shared equally; that there was equal benefit for the Nepal and U.S. team members (Nepal, 58.3%, n = 7; U.S., 66.7%, n = 6); and that they made decisions together (Nepal, 69.2%, n = 9; U.S., 55.6%, n = 5) (see Fig 5). When asked, “How well did the U.S. team understand and respect Nepali culture?” all (n = 13/13; 100%) of the Nepal survey respondents answered “The U.S. team understood and respected Nepali culture most/all of the time.”

#### Impact of COVID-19

The COVID-19 pandemic affected both the U.S. and Nepal team members, but to different degrees. The majority of U.S. respondents (77.8%, n = 7) indicated the pandemic affected their individual ability to complete work on the project only “a little” (22.2%, n = 2) or “not at all,” (55.6%, n = 5). In contrast, 77% (n = 10) of Nepal respondents reported the pandemic affected their individual ability to complete work on the project “somewhat” (46.2%, n = 6) or “a lot” (30.8%, n = 4). On a group level, 46.2% (n = 6) of Nepal respondents indicated the Nepal group was “somewhat” affected by COVID-19; U.S. respondents indicated that COVID-19 impacted their group either “a little” (33.3%, n = 3) or “not at all” (22.2%, n = 2). The difference between the Nepal and U.S. groups related to “how much did COVID-19 impact your personal/individual ability to complete work?” was statistically significant (p = 0.01).

**Free-text responses.** Our survey included 6 free-text items where respondents were invited to provide more in-depth responses (Table 2; see also S1 Text).

**Table 2:** Summary of free-text survey responses.

What is the most important thing you learned working on this project?
• I learned the complexity of conducting research in a limited resource setting and how to address those challenges successfully. *(U*.*S*. *Participant)* • Working with the NIH grant is new experience, and I learned the various aspects of the scientific and financial management of the project. *(Nepal Participant)* • Teamwork, new ideas, work ethics, newer content on research, networking. *(Nepal Participant)* • Learned the way of communication, research skills, way of collecting data, time management skills, [how to] develop good personal relation within the team member. *(Nepal Participant)* • I learned much about the need for building research capacity in low- and middle-income countries. It was such a pleasure meeting regularly with the Nepal team, and the leadership team did an outstanding job. *(U*.*S*. *Participant)* • Appreciation for a different culture. *(U*.*S*. *Participant)*
**Is there anything you wanted to learn during the project, but did not get the chance to learn?**
• I wanted to learn more technical knowledge of each step of the research process but did not get the chance to because of limited time related to work and school. *(U*.*S*. *Participant)* • Financial proposal and effective utilization of budget. *(Nepal Participant)* • Yes, about the grant writing. *(Nepal Participant)*
**What did you like best about participating in this project?**
• Witnessing the research process in person through the workshop. *(U*.*S*. *Participant)* • Collaborating with colleagues from Nepal and knowing that the project was contributing to improved patient care. *(U*.*S*. *Participant)* • Teamwork and the various phases of the research project where we work for questionnaire development, mobile app development and virtual library. *(Nepal Participant)* • The Nepal and U.S. team. Everyone was so nice and wanting to help each other out. It made me excited to go to the Zoom meetings. *(U*.*S*. *Participant)* • Best part was that we were completing this project with new energy and with very joyful manner without stress and burden. *(Nepal Participant)* • Working with a fantastic, dedicated, kind team! *(U*.*S*. *Participant)* • The collaborations. Learning from the Nepal team. Everyone had such a positive attitude and were very supportive of each other. So, really, it was the teamwork that I liked best. *(U*.*S*. *Participant)*
**What did you like least about participating in this project?**
• Having not enough time to participate in this research. *(U*.*S*. *Participant)* • I was only involved in latter part of the project. Probably if COVID was not there, would like to work some of it in person. *(Nepal Participant)* • Logistical and political challenges. *(U*.*S*. *Participant)* • The distance between this project’s work and actually improving patient’s lives felt pretty far to me. *(U*.*S*. *Participant)* • The physical distance between the teams. *(Nepal Participant)*
**What activity did you find most meaningful or helpful?**
• The workshop and the in-person involvement in the data verification. *(U*.*S*. *Participant)* • Development of mobile app and virtual library. *(Nepal Participant)* • Project proposal revision and various steps of virtual library development where we learned many things in simple way. Here learning was fun. *(Nepal Participant)* • Thinking process, work ethics, cultural respect, tendency to help people of cancer with pain, a holistic approach. *(Nepal Participant)* • I think visiting Nepal and getting to be with the team members in person was most helpful. Something about that person-to-person contact, being able to drink tea and eat cookies and chat during the breaks, really helped to build goodwill and better relationships, which helped me to feel more invested in the project as a whole. *(U*.*S*. *Participant)*
**Anything else you’d like to share about your experience?**
• This was a great opportunity for me as a student to be able to participate in research. The timing couldn’t have been better because I had just completed a course in research and statistics which helped me witness the different steps of research in real life. *(U*.*S*. *Participant)* • Grateful to have been a part of such a wonderful team that truly embraced the team spirit. *(U*.*S*. *Participant)* • It was really an honor to be able to participate. I attribute this positive experience to having been able to work with really good people on the project. *(U*.*S*. *Participant)* • I had a great opportunity to have experience to be part of NIH grant and networking with various colleagues and researched within and in USA was an important aspect. *(Nepal Participant)*

### Interview results

#### Semi-structured interviews

**Fig 6:**
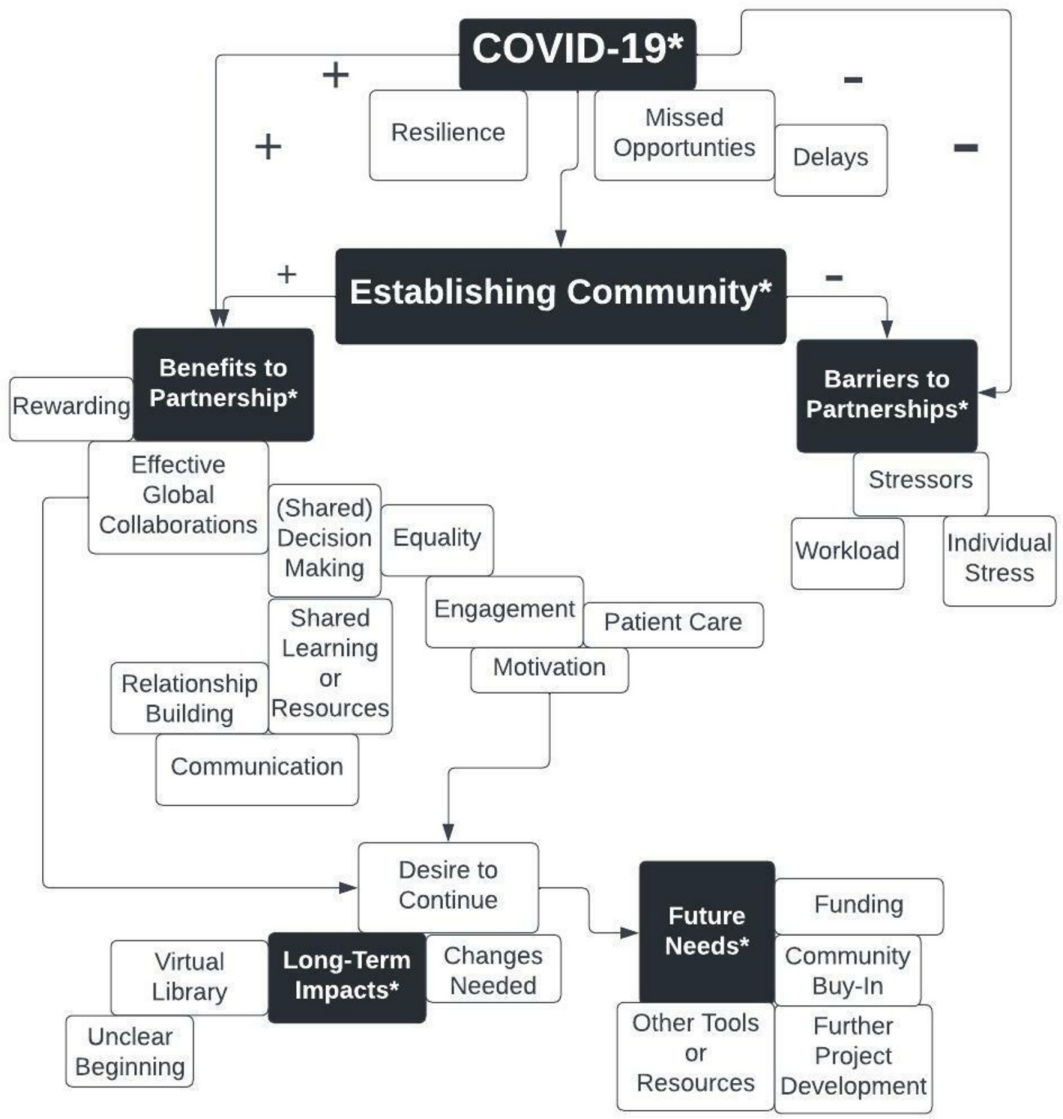
Code map based on qualitative analysis of semi-structured interviews.

Of the 22 survey respondents, 8 (n = 8, 36%) agreed to be interviewed, 4 (n = 4; 50%) from the U.S.; 4 (n = 4; 50%) from Nepal. The over-arching theme that emerged from the interviews was the importance of ‘establishing community,’ manifested primarily by forming connections within, and across, groups. Participants discussed the theme of ‘establishing community’ within the broader context of COVID-19, specifically in how the pandemic profoundly influenced developing a community, in both positive and negative ways. Given the prominence in which the pandemic was discussed by participants, ‘COVID-19’ was placed at the top of our qualitative code map to represent its significant impact (see Fig 6), in ways that were both constructive (represented by a “+”) and less-constructive (represented by a “-”). Additional key themes (represented with asterisks, Fig 6) included the benefits and barriers to global partnerships, long-term impacts, and future needs. Categories supporting each of these themes include the project’s focus on shared learning; individual stressors; sustainability; and the need for community buy-in. Illustrative quotes that support key themes are summarized below and also within Table 2.

*The impact of COVID-19 in establishing community*. The COVID-19 pandemic occurred midway through the project and its impact included the cancelation of planned travel and dissemination opportunities, missed opportunities for in-person connection, and project delays. This was felt across both groups, as noted by U.S. Participant 4: “There were trips planned, and I think that would have just really strengthened the project further. A lot of these projects, and this is true for international work in general, require that face-to-face and doing things on Zoom is just not the same as being there in person, no matter how hard we might try. [But Zoom] certainly allowed the project to continue.” A similar sentiment was expressed by Nepal Participant 2: “[The project] takes a longer time due to unavailability of various resources like bank closings and closing of the training programs. We had to extend this project a little bit more.”

The stress of the pandemic was also experienced on a more personal level, that extended beyond the research aims of the project. For example, Nepal Participant 3 said, “[There was a] great stress scenario like what will be done next, like how their life will change by this COVID…psychologically majority of professionals are also get burdened. This may have affected this project.” Several participants commented on the relative resilience of the project in spite of the pandemic and discussed that the imposed isolation was somewhat mitigated by the opportunity to virtually connect with team members across the globe. For example, from Nepal Participant 4: “We might be oceans apart, or continents apart, but…even during this pandemic times nothing stopped, for us…That goes to show the human resilience and commitment towards a better world.” Additionally, U.S. Participant 4 commented, “I think also especially with COVID, I felt kind of isolated, you know? Just always being in my apartment. And then like, ‘Oh, I get to meet people that live on the other side of the world. I’m still meeting people even though there’s COVID.’”

*Benefits to partnership*. Overall, a positive experience was reported by all interviewed participants. Even within the broader context of working on the project during the profound stressors of COVID-19, participants still felt community was productively established and described it as the key benefit to the partnership. Establishing community is considered critical to building research capacity at all levels of the Social Ecological Model [33]. Perceived benefits of the collaboration were experienced by participants related to shared decision-making, shared learning, communication and ensuring equitable engagement (see Fig 6 and Table 3). For example, Nepal Participant 4 commented:

“So, it was, realistically speaking, a very collaborative effort, you know? And everyone from the UVA team treated us equal, and so did we. […] Contribute as equals and share our ideas, which are equally respected.”

**Table 3:** Additional supporting quotes from semi-structured interviews.

Themes and Categories	Supporting quotes
**COVID-19**	
Missed Opportunities	“So, I never actually met anybody in person…Yeah, it was a little sad that I know they wanted to do like trips to Nepal, and everything.” (U.S. Participant 4) “You can do a lot virtually and we did pretty good, but there’s something about meeting each other in person and like having coffee with them during the lunch break, or like, you know, chatting with them about their family.” (U.S. Participant 2)
Delays	“And then certainly from just like a logistics perspective, paying for things is just, it’s hard. Like banks shut down in Nepal. We couldn’t send money, and then they couldn’t get the money that was sent. And people had to wait for a long time to pull money out and to pay people. And so that’s hard. To get their money for the work that they’re doing! And, you know, a lot of that was really delayed.” (U.S. Participant 1)
**Benefits to Partnership**	
Shared Decision Making	“[…] we were doing the readings…and we were like switching with our peers whether what we were thinking [among our groups was correct and] we were trying to see things from lots of angles.” (Nepal Participant 3)
Shared Learning or Resources	“I think it’s really a bi-directional benefit […] certainly the high resource setting has perhaps experience and expertise that they can offer to the lower resource setting, in this case, our Nepal colleagues. But I also think that we certainly learned a tremendous amount from the Nepal colleagues.” (U.S. Participant 3)
Communication	“The first thing is that there is very good communication between the teams. I was entered in the middle of the project […] I was given instruction, clearly about all those processes.” (Nepal Participant 1)
Equality	“I think everyone was kind of learning from each other. I learned a lot from them. I also learned a lot from [our] team.” (U.S Participant 4)
**Barriers to Partnerships**	
Stressors	“Like banking systems. . .that was a huge thing for this project, in particular that was really challenging […] learning how to navigate different banking systems and their rules and requirements, and the amount of work that that involved on an individual level to make [it] happen.” (U.S. Participant 1)
Individual Stress	“I also think there are things like cultural barriers, language barriers, just not understanding the system. So, there was a big learning curve, for me, in terms of just understanding, like the four hospital clinic sites that we were looking at, you know? I couldn’t, I had a hard time keeping their names straight, much less like understanding what was unique about those places qualitatively.” (U.S. Participant 2)
**Long-Term Impacts and Future Needs**	
Continued Funding	“I think it’s hard when these things end. Because you’re like, "Oh, we’ve done such great work! Let’s keep going!" And then, "Oh, wait." Pause. And then we can continue. I have hopes and I’m optimistic that it will. It will continue, for sure. I just think we just got to get the money. It’ll happen.” (U.S. Participant 1)
Community Buy-in	“But I think now we have understood how it’s going to come. I think slowly the level of research has to go, step by step, maybe in another project, which is better than the first one […] I think the level has to keep on going up and up.” (Nepal Participant 3)
Further Project Development	"So, continue to have the research will be better than developing another app in the particular setting because if we did not do any follow up of this project […] we will not be informed […] about the effectiveness of the app development.” (Nepal Participant 2)

U.S. Participant 2 also reflected on the mutual benefit of the project and the importance of utilizing a participatory approach:

“But this also seemed a little bit different because [U.S. PI] had been collaborating with some of the members of this team for years if not decades. I also knew going into it that there was a lot of bi-directional [work]. Like the work wasn’t just being dictated by [U.S. PI] as the outsider of sort of this partnership […] they [Nepal team members] were like, ‘We have this issue, and we want to work on it together.’” (U.S. Participant 2)

Additionally, a U.S. Participant 4 (a student) shared a potential benefit to the shared learning and decision making of the project in its ability to improve patient care:

“I feel like, ‘Oh, this is actually going to impact people.’ Like it’s not just, ‘I’m getting this one assignment and my professor is going to see it and then we’re done.’”

Nepal Participant 1 echoed how this collaborative research could benefit HCP in Nepal:

“[…] the majority of the healthcare professionals are not training [in] palliative care and pain management, but we hope that this app [one deliverable from the parent project [26]] will help them to treat the patient and to manage it.”

When reflecting on communication, the importance of role clarity to foster a productive collaboration was emphasized. For example, U.S. Participant 3, reflected on the importance and success of, “…having an agreement on what’s expected in terms of roles and responsibilities throughout the collaboration, but then also that setting up the regular communication and staying in touch and keeping each other updated.” This was especially essential during COVID-19, where online meetings were often the only way to communicate and required consistent scheduling and regular participation. Nepal Participant 1 noticed this did not diminish during the pandemic:

“Like we don’t have to be burdened due to the distance. We have the instant messages; we can get a message in the WhatsApp to the team members from the U.S. and Nepal, too. So even though we do not, we couldn’t meet physically but, personally, we are getting the communication better.”

Ensuring equitable engagement was important to both groups and felt to be a critical ingredient to ensure a beneficial partnership. U.S. Participant 2 emphasized the importance of inclusion across team members:

“I remember saying to [the team] that [name of Nepali RN] didn’t speak during this meeting, and I really want to know what they think about this [issue]. I felt like we needed to put in a little bit of extra effort to hear from every member of the team, especially those that might have been less likely to speak up in a group setting.”

Nepal Participant 4 also reflected on this effort towards inclusion, “…everyone took the time and the effort and everyone was very receptive.”

*Barriers to partnerships*. Barriers to this partnership were discussed less than perceived benefits, but manifested in the form of specific stressors, such as learning new systems or navigating relational experiences (see Table 3). Overall, Nepal team members were quick to dismiss negative experiences, even with follow-up questions (see S2 Text) and were reframed as positive. For example, when asked if the equality between groups was, at any point, out-of-balance, a Nepal participant explained:

“No, I was so happy with the U.S. team. I’m not just saying this because I’m on record […] I think what is most required in any relationship is mutual trust, understanding and respect. And that was there. That was there.” (Nepal Participant 4)

However, Nepal team members did highlight a few concerns about global partnerships, in general, including the practice of assuming that what works in higher-resourced settings is appropriate for potentially lower-resourced contexts, and emphasized the importance of inclusion of LMIC participants in data creation.

“[…] what’s the saying that these researches happen in the West. And same data applies for people in the East. That is what usually happens, you know? […] We usually have guidelines that are mostly based on the West. Now, do we apply that to our countrymen or country fellow persons? Now that is a questionable thing.” (Nepal Participant 4)

“[…] we need to generate our data ourselves to make it real.” (Nepal Participant 3).

Another Nepal participant also emphasized the need for communication transparency, especially around funding and financial matters: “Because once the money goes to the organization, they do not have the clear guideline how they can disseminate to the team, right? […] it should [formally] be approved from that organization and submit the bills the proper way.” (Nepal Participant 2)

*Long-term impacts and future needs*. Sustainability of the project focused on the perceived long-term impact of the project, such as the Virtual Library [27] as well as future needs, which included continued funding, community buy-in and a need for further project development that builds upon prior work (see Fig 6 and Table 3).

Generally, participants were hopeful and conveyed an eagerness to continue with the collaboration and its work:

“So, well I would wish that it continues, and I can only speak for myself. That I would definitely do it, even if it is volunteer.” (Nepal Participant 4).

Others spoke to the benefits of an established relationship that would hopefully increase the chances of a future, on-going partnership:

“I think that another impact would be the sort of establishment and enhancement of this partnership with Nepal […it] lays a foundation for more work in the future. Which is very important.” (U.S. Participant 3)

## Discussion

Overall, this multi-method evaluation of a global health collaboration between partners in Nepal and the U.S. revealed strong bi-directional benefit and a greater emphasis on perceived benefits versus challenges, despite the additional stressors of COVID-19. In both the quantitative surveys and the qualitative interviews, the majority of participants reported that power was shared equally, there was equal and mutual benefit for all team members, decisions were made collaboratively, and both groups worked well together. Almost 100% of respondents (all but one participant, who answered ‘sometimes’) reported “yes, always” when asked if their voices and opinions were heard and respected during the project. Both our survey results and semi-structured interviews contribute to, and extend, the existing research capacity-building literature by offering perspectives from a diverse interdisciplinary team from both high and low-resource contexts that can benefit others engaged in similar global public health work. We also offer an exploration as to how research capacity collaborations may be impacted during times of significant global stress, such as a pandemic. Our survey tool and interview guide, intentionally designed to comprehensively examine the impact of a global partnership across various levels of the Social Ecological Model [33] (individual, institutional, country/system), can be adapted and used to evaluate other similar research capacity collaborations.

Our electronic survey had a good response rate for email based surveys [42], 52.4%, which may reflect, at least in part, the strong investment team members had in the project and collaboration. Overall, our survey respondents, were primarily female-identifying (68%), providing another important contribution to the research-capacity building literature [15]. There were some important differences between team composition that likely influenced survey results. The Nepal team was composed entirely of actively practicing HCPs, most having over 10-years of direct patient care experience (92.3%), and the majority being physicians (69.2%). This reality likely explains higher ratings of the impact of COVID-19 by Nepal team members, all of whom were delivering front-line pandemic care in severely stressful and resource-constrained contexts during the collaboration [43]. In contrast, the U.S. team was less clinically focused, included undergraduate and graduate students, had enhanced flexibility and infrastructure to support remote work from home, and were the beneficiaries of earlier vaccine availability. All of these factors likely influenced a lower perceived impact of the pandemic on the project by U.S. team members. The presence of students on the U.S. team limited robust responses related to survey items related to the budget or finances, as no students were directly involved with fiscal administration of the project. The number of students on the U.S. side may also explain the lower ranking of ‘career advancement’ and/or ‘publication opportunities’ as a key motivator for joining the project. Additionally, the difference between team members regarding role clarity (e.g., less Nepal team members strongly agreeing that their individual roles were clear) may be due to a higher number of U.S. team members with more experience in research and its associated tasks, as well as the higher number of U.S. based closely mentored undergraduate and graduate students. Another possible explanation is that not all Nepal survey respondents and interviewees were involved in the earliest stages of the project and proposal development, as many of them joined the project after its first year.

We are particularly encouraged that self-reported confidence and experience with research increased after participation in the project for both Nepal and U.S. team members and that both groups reported high levels of ‘overall learning’ from the project (i.e., the majority of Nepal team members indicating “a fair amount” of learning (76.9%, n = 10) and U.S. team members indicating “a lot” (62.5%, n = 5). Increases in “confidence with research,” along with “overall learning” were statistically significant between groups, despite a small sample size, suggests the importance of including these variables in evaluating the impact of global research collaborations. One possible interpretation of this difference, offered by our Nepali colleagues, may be related to a growing culture of research within Nepal, with more HCPs seeking out research opportunities as a learning platform. Another explanation may be the higher number of students on the U.S. team, who generally entered the project with very little knowledge of research and so had great capacity for growth.

When comparing responses to questions about relationship building, sustainability, and having the resources and/or funding necessary to complete the project, both surveys and interviews converged on a primarily positive outlook. For example, all team members from both groups agreed they had the resources needed to complete work on the project, and 69.2% (n = 9) of Nepal team members and 66.7% (n = 6) of U.S. team members “strongly agreed” that the project was likely to continue on in the future. Compared to Nepal team members, more U.S. participants either “strongly agreed” or “weren’t sure” when asked how well budget and financial issues were handled during the project, which likely reflects that the U.S. group included both individuals highly involved with the budget as well as those with no involvement (e.g., students).

Financial and budget matters were important topics in both survey and interview results. These issues can be particularly complex in global collaborations, as differing cultural norms related to salaries and supplemental fees, banking practices and documentation, and reliance on cash-based transactions, all can vary across different countries and cultures. Managing finances for the project was one of the most complicated and time-consuming aspects of the project (both logistically and politically) but impacted a smaller number of team members who were directly involved in the fiscal administration of the project. For these individuals, the pandemic further complicated the already complex financial management of this project due to bank closures that made it impossible to transfer/wire money, distribute funds, and make timely payments.

Mutual benefit to both parties is emphasized as important in current research capacity-building literature [15], and overall this was demonstrated in our results. There were some slight differences, however, in survey item responses related to team member perceptions of shared understanding of project goals, equal benefit, and power distribution, with fewer Nepal team members answering ‘strongly agree” compared to U.S. team members. One potential reason for this could be related to variability in how respondents interpreted specific survey items. For example, did every participant answer these questions with the same operational definition or cultural understanding of “shared decision making” or what was meant by “power?” We, unfortunately, did not define these concepts within the survey as we did for “research capacity,” so interpretation could have been variable. Future evaluations should clearly define such terms and phrases, especially in asynchronous surveys or quantitative evaluations where there may not be a face-to-face opportunity for clarification between researcher and participant. Students and others who entered the project midway may also had not scored “shared decision making” or “power being shared equally” highly simply because they had less experience with the project and team. Additionally, differences in responses may be related to a cultural reluctance to select extremes on a Likert scale-based survey item (e.g. collectivist cultures may be less likely to select “strongly agree” than more individualistic cultures [44]); this was not borne out in the interviews.

An important contribution of our evaluation is assessing the project’s perceived effect on research capacity and delivery of care at the individual, institutional, and country/system level. Overall, team members rated the project’s impact favorably across the Social Ecological Model, with over 60% of respondents either ‘agreeing’ or ‘strongly agreeing’ with all statements. However, Nepal respondents were less likely to ‘strongly agree’ with some statements compared to U.S. respondents, and this difference was most striking around the question, “the project will help providers in Nepal deliver quality cancer care/palliative care” with the Nepal team ‘strongly agreeing’ (30.8%) compared to the U.S. (77.8%). This could be due to the composition of the Nepal survey respondents (i.e., all health care providers) who possessed a greater and more realistic knowledge of research capacity growth challenges within Nepal compared to U.S. survey respondents who perhaps overestimated the project’s impact, being less familiar with the context, care delivery and set-up in Nepal. Other explanations include how questions related to patient care were interpreted by Nepali colleagues in the context of COVID-19 (which our parent project was not designed to address), or, as mentioned above, due to a general reluctance of Nepali colleagues to select the ‘extreme’ responses (e.g., strongly agree or strongly disagree) on the Likert-scale questions [44]. While further consideration and mitigation of these differences is warranted, we are encouraged that that no Nepali respondents indicated ‘somewhat disagree’ or ‘strongly disagree’ in any category. In other words, *all* Nepal team members agreed (either ‘somewhat agreed’ or ‘strongly agreed’) that the project had improved research capacity across all levels of the Social-Ecological Model. We feel this is especially significant given the fact this collaboration spanned multiple years, a global pandemic, diverse cultures, and differing baseline research infrastructures.

During the interviews, “building community” and “benefits to partnership” were prominently discussed by all participants. We were surprised by the overwhelmingly positive responses in the interviews, so much so we made an especial effort to probe and understand what may not have gone well. Interestingly, even these follow-up questions generally resulted in barriers or potential negative aspects being spun or reframed in a positive light. One interpretation of this is that our collaboration really did excel in all areas. A much more likely explanation is that participants were hesitant to speak negatively about the project due to cultural communication norms, fear of offending colleagues, or a pragmatic understanding that future funding (and financial incentives) may be tied to a positive evaluation of the project. It is also likely that our interviews self-selected for team members with a particularly positive experience they were eager to share. Regardless, these interviews provided important independent insights and allowed us a greater opportunity to explore the impact of COVID-19.

The impact of COVID-19 was noted with survey responses and interviews, where frequent and spontaneous discussion of the topic occurred. For example, participants often described delays and missed travel opportunities directly related to COVID-19 when asked about the project’s general timeline or would reflect on how COVID-19 shifted the project’s context to an even stronger reliance on technology (e.g., Zoom) once the pandemic took hold. It is important to note that the use of technology has long been considered a key domain for facilitating research capacity [15]; our collaboration during reinforced this importance many times over. Other significant pandemic impacts included professional and personal stress (particularly for frontline HCPs in Nepal), country-based policy changes, and the extent and timing of “lockdowns.” Remarkably, and in spite of these challenges, participants conveyed positive anecdotes, including the dedication of team members, the project’s general success, and strong hopes for moving forward. When considering survey results, a statistically significant difference was found between the Nepal and U.S. related to “how much did COVID-19 impact your personal/individual ability to complete work?” (p = .01). As noted, this difference between groups could be tied to demographic differences, as all of the Nepal team members identifying as active HCPs. But, more critically, these findings also serve as an important reminder of the persistent inequities between HIC and LMIC in coping with a global health emergency.

## Limitations

The primary limitation of this study is our smaller sample size which necessitates caution in interpreting statistical results and our focus on one specific global partnership, which can limit generalizability. However, our sample size was proportional to the overall study team membership and we had a strong survey response rate for email based surveys [42], and an appropriate sample size for descriptive qualitative research [45]. We also suggest that while this project focused on two particular countries (Nepal and the U.S.), the primary findings and the comprehensively developed, evidence-informed framework for how to evaluate the project could be applied to similar global health and research capacity building collaborations. An additional potential limitation could be social desirability bias (i.e., respondents reporting more favorable views of the project to satisfy perceived preference of the investigator). We strove to mitigate this by having a neutral party (VA), who was not affiliated with the parent study, send out survey and interview invitations and conduct semi-structured interviews and by utilizing both quantitative and qualitative data collection methods. Our surveys assessed respondents at the end of the project, and therefore any respondent baseline (‘pre’) self-rankings were retrospective versus recorded *a priori*. Further, while we invited all team members to participate in the project evaluation it is important to acknowledge that members (both U.S. and Nepal) had varying roles and levels of engagement throughout the multi-year research collaboration and joined the project at different times; this reality likely influenced results and perceptions of participants.

It is also important to note that while we utilized a multi-method approach, and intentionally use this terminology to describe our study design, the smaller sample size precludes robust statistical analysis and integration of quantitative and qualitative findings that characterizes a traditional ‘mixed methods’ study. While we strove to utilize the interviews to better contextualize the survey findings, we found participants chose to discuss different topics, in different ways, during the interviews. For example, the impact of the COVID-19 pandemic on the collaboration was a more prevalent theme during interviews than was revealed within the surveys). Another limitation in using the interviews to fully contextualize our survey findings is that not all survey respondents agreed to participate in an interview and because our surveys were anonymous we could not link survey responses to specific interview participants.

## Conclusion

Comprehensively evaluating research collaborations is critical to ensure global public health projects are optimally effective and productive. In this paper, we present results from our own project evaluation and offer our survey and interview guide as an evaluation ‘toolkit’ that we hope will be useful to others engaged in similar work to help promote equitable and sustainable public health collaborations. Future evaluations of global research-capacity building projects should consider the benefits of a multi-method approach that allows for more anonymous survey feedback (where respondents may be more ‘honest’) augmented with qualitative interviews (where participants may respond more ‘fully’). We found utilizing a multi-method approach to explore a project’s impact across all levels of the Social Ecological Model from both LMIC and HIC partners beneficial. In our evaluation of our Nepal-U.S. based research capacity building partnership, we identified many strengths, but also opportunities for improvement to develop collaborations that are truly equitable–an on-going and continuous goal that must supersede the specific aims of any particular project.

## Supporting information

S1 Text UVA/Nepal research partnership survey. (DOCX)

S2 Text Semi-structured interview guide. (DOCX)

S3 Text Global inclusivity questionnaire. (DOCX)
