# Supplementary material for: Co-constructing collaboration: An evidence-based approach to advance and evaluate equitable global public health research partnerships
Source: PLOS Glob Public Health. 2023 Oct 23;3(10):e0002481. doi: 10.1371/journal.pgph.0002481 (PMC10593218; doi:10.1371/journal.pgph.0002481)
Supplement: S1 Text — (DOCX) [file pgph.0002481.s001.docx]

S1 Text

UVA/Nepal Research Partnership Survey

Part 1.  (10 questions) Demographics
The first part of this survey asks questions about yourself.  There are no right or wrong answers!   All answers are anonymous and will not be linked to you.

1. What is your age?

1. 18- 30 years old
2. 31- 40 years old
3. 41- 50 years old
4. 51- 60 years old
5. Over 60 years old

2. What is your preferred gender identity?

a) Male

b) Female

c) Other

3. Are you trained as a healthcare provider?

1. Yes
2. No

If a respondent answered “yes” to question 3, the following questions followed. If they answered “no” the respondent was skipped to question 6:

4. What type of healthcare provider are you?

1. Nurse
2. Physician
3. Other

5. How long have you been a healthcare provider?

1. Less than 5 years
2. 6 - 10 years
3. 11 - 20 years
4. 20 years or more

6. How much of your current job is spent providing **direct patient care?**

1. 0 - 25%
2. 26 - 50%
3. 51 - 75%
4. 76 - 100%

7. How much of your current job is spent **conducting or supporting research?**

1. 0 - 25%
2. 26 - 50%
3. 51 - 75%
4. 76 - 100%

8. Were you in a student role (undergraduate or graduate) on this project?

1. Yes
2. No

9. Were you in a faculty member role (non-student) on this project?

1. Yes
2. No

10. For this research project, are you a member of the **Nepal team** or the 
**UVA (non-Nepal)** team?

1. Nepal
2. UVA (non-Nepal)

**Part 2. (13 questions)**
The next set of questions asks you **about your experiences**working with this research project. There are no right or wrong answers!   All answers are **anonymous** and will not be linked to you.

1. How long were you involved with the Nepal / UVA research project, in any capacity?

1. Less than 6 months
2. 6 months - 1 year
3. 1 - 2 years
4. More than 2 years

2. **Overall**, how would you **rate your experience** participating in this research project?

1. Very negative
2. Somewhat negative
3. Neutral: Neither negative or positive
4. Somewhat positive
5. Very positive

3. What did you do during the research project?
 **Please select all that apply**

- I helped develop and submit grant proposals to the National Institutes of Health (NIH)
- I helped design the pain management survey (the first survey)
- I collected data for the pain management survey (the first survey)
- I analyzed data for the pain management survey (the first survey)
- I was a co-author on an abstract (poster or podium) related to the pain management survey
- I was a co-author for the pain management survey manuscript for publication
- I helped build/design the NAPCare Mobile Application
- I helped design the mobile app beta testing survey (the second survey)
- I collected data for the mobile app beta testing survey (the second survey)
- I analyzed data for the mobile app beta testing survey (the second survey)
- I was a co-author on an abstract (poster or podium) related to the mobile app testing
- I plan to be a co-author for the mobile app beta testing manuscript for publication
- I helped build/design the Virtual Library
- I helped find and review content for the Virtual Library
- I analyzed data related to the Virtual Library
- I was a co-author on an abstract (poster or podium) related to the Virtual Library
- I plan to be a co-author for the Virtual Library manuscript for publication
- I participated in the first UVA-Nepal workshop in Nepal (January 2019)
- I participated in the second UVA-Nepal workshop in Nepal (July 2019)
- I participated in the third UVA-Nepal workshop in Nepal (January 2020)
- I participated in the first Nepal Health Research Training workshop (June 2021)
- I participated in the second Nepal Health Research Training workshop (July 2021)
- I regularly participated in team meetings over Zoom
- I supervised research team members to ensure tasks were completed
- I mentored team members to develop their research skills
- I helped organize and plan administrative details of the research project

4. **Overall**, how much do you feel you **contributed** to the project?

1. Nothing
2. A little
3. A fair amount
4. A lot

5. **Before** this project, how much **experience** did you have with research?

1. None
2. A little
3. A fair amount
4. A lot

6. **After** this project, how much **experience** do you have with research?

1. None
2. A little
3. A fair amount
4. A lot

7. **Before** this project, how much **confidence** did you have with research?

1. None
2. A little
3. A fair amount
4. A lot

8. **After** this project, how much **confidence** do you have with research?

1. None
2. A little
3. A fair amount
4. A lot

9. **Overall**, how much **did you learn** about research during this project?

1. Nothing
2. A little
3. A fair amount
4. A lot

10. What **motivated** you to participate in this research project?
**Please select all that apply**

- Develop and learn skills
- Career advancement
- Publication opportunities
- Opportunity to participate in future similar projects
- Mentorship opportunities
- Encouraged to participate by supervisors
- Increase job satisfaction
- Financial incentive
- Improve patient care
- Keep the brain stimulated
- None of the above / I had no motivators
- Other (please write in) __________________________________________________

11. What were **barriers** for you to participate in this research project?
**Please select all that apply**

- Other work roles taking priority
- Lack of time for research
- Lack of research skills
- Lack of funding
- Lack of staff
- Lack of equipment
- Limited library access
- Little access to required software
- Poor access to Internet service
- Other personal or family commitments
- COVID-19 affecting my job / my co-workers
- COVID-19 affecting me / my family personally
- None of the above / I had no barriers
- Other (please write in) __________________________________________________

12. On **average**, how much time per week did you spend working on this project?

1. Less than 2 hours per week.
2. 2 - 4 hours per week.
3. 4 - 8 hours per week.
4. More than 8 hours per week.

13. Was the **amount of time** spent working on the project what you expected?

1. It was **less time** than I expected.
2. It was **about the amount of time** I expected.
3. It was **more time** than I expected, **and this was okay.**
4. It was **more time** than I expected, **and this was a problem.**

**Part 3. (20 questions)**
The next set of questions ask about **research capacity**. For this survey, **research capacity** is defined as "the ability to engage in, perform, or carry out quality research." (Matus et al., 2018). There are no right or wrong answers!  All answers are **anonymous** and will not be linked to you.

1. How much do you agree with these statements:

|  | Strongly Disagree | Somewhat Disagree | Neutral / Unsure | Somewhat Agree | Strongly Agree |
| --- | --- | --- | --- | --- | --- |
| This project improved research capacity for me as an **individual**. |  |  |  |  |  |
| This project improved research capacity for my **organization/institution**. |  |  |  |  |  |
| This project improved research capacity for **Nepal**. |  |  |  |  |  |
| This project improved/will improve the lives of **patients** with cancer **in Nepal.** |  |  |  |  |  |
| This project improved/will improve the lives of **patients** with cancer in **other low and middle-income countries**. |  |  |  |  |  |

2. How much do you agree with these statements:

|  | Strongly Disagree | Somewhat Disagree | Neutral / Unsure | Somewhat Agree | Strongly Agree |
| --- | --- | --- | --- | --- | --- |
| This project helped/will help **healthcare care providers** **in Nepal** deliver quality cancer care/palliative care. |  |  |  |  |  |
| This project helped/will help **healthcare providers in other low and middle-income countries** deliver quality cancer care/palliative care. |  |  |  |  |  |
| This project is **likely to continue forward** in the future. (e.g. is **sustainable**). |  |  |  |  |  |

3. How would you rate the **frequency** **of communication** between the UVA and the Nepal team during this research project?

1. There was **too little** communication.
2. There was **about the right amount** of communication.
3. There was **too much** communication.
4. I'm not sure.

4. How would you rate the **clarity** **of communication** between the UVA and the Nepal team during this research project?

1. Communication was **very clear.**
2. Communication was **somewhat clear.**
3. Communication was **not clear at all.**
4. I'm not sure.

5. If you had **concerns or questions** about the project, did you feel you had someone to talk to?

1. Yes, always
2. Sometimes
3. No, never
4. I'm not sure

6. Do you feel your voice and opinion was **heard and respected** during the research project?

1. Yes, always
2. Sometimes
3. No, never
4. I'm not sure

7. Was there was **hidden or open conflict** among team members that made it difficult to complete the project?

1. Yes, there was some conflict **and** **it made it difficult** to complete the project.
2. Yes, there was some conflict **but it did not** make it difficult to complete the project.
3. No, **there was no** conflict.
4. I'm not sure.

8. If / when conflicts occurred, did the UVA and Nepal teams **work together** to resolve them?

1. Yes, the teams **always** worked together to resolve conflict.
2. Yes, the teams **sometimes** worked together to resolve conflict.
3. No, the teams **did not** work together to resolve conflict.
4. I'm not sure.
5. I do not feel there was any conflict.

9. How much do you agree with these statements:

|  | Strongly Disagree | Somewhat Disagree | Neutral / Unsure | Somewhat Agree | Strongly Agree |
| --- | --- | --- | --- | --- | --- |
| I had the **resources** I needed to complete work on this project. |  |  |  |  |  |
| The **overall goals** of this research project were clear to me. |  |  |  |  |  |
| **My role and what was expected of me** with the project was clear. |  |  |  |  |  |
| I felt **supported** in my work on this project. |  |  |  |  |  |

10. How well were **budget / financial issues** handled during the project?

1. Budget / financial issues were handled **very well.**
2. Budget / financial issues were handled **somewhat well.**
3. Budget / financial issues were handled **poorly / not well at all.**
4. I'm not sure.

11. How much do you agree with this statement:
 This project demonstrated effective use of financial and other resources.

1. Strongly Disagree
2. Somewhat Disagree
3. Neutral / Unsure
4. Somewhat Agree
5. Strongly Agree

12. How much do you agree with these statements:

|  | Strongly Disagree | Somewhat Disagree | Neutral / Unsure | Somewhat Agree | Strongly Agree |
| --- | --- | --- | --- | --- | --- |
| The UVA and Nepal team **made decisions together** on this project. |  |  |  |  |  |
| The UVA and Nepal teams **had a shared understanding** of the project goals, priorities and strategies. |  |  |  |  |  |
| There was **equal benefit** for the Nepal team and UVA team with this project. |  |  |  |  |  |
| **Power was equally shared** between the Nepal team and the UVA team during the project. |  |  |  |  |  |
| Goals for the project were **mutually identified and agreed upon** by the Nepal and UVA team during the project. |  |  |  |  |  |

13. How would you rate the **level of trust** between UVA and Nepal team members during this project?

1. No trust at all
2. A little trust
3. A fair amount of trust
4. A lot of trust
5. I'm not sure

14. How much do you agree with this statement:
 **Overall**, this project had effective leadership.

1. Strongly Disagree
2. Somewhat Disagree
3. Neutral / Unsure
4. Somewhat Agree
5. Strongly Agree

15. How much do you agree with this statement:
**Overall**, the UVA and Nepal teams worked well together.

1. Strongly Disagree
2. Somewhat Disagree
3. Neutral / Unsure
4. Somewhat Agree
5. Strongly Agree

16. Did this project help team members **grow and learn** from one another?

1. Yes, there was **growth for everyone.**
2. There was **growth for some, but not for all.**
3. No, there was **no growth for anyone.**
4. I'm not sure.

17. How well did the UVA team **understand and respect** Nepali culture?

1. The UVA team understood and respected Nepali culture **most / all** of the time.
2. The UVA team understood and respected Nepali culture **some** of the time.
3. The UVA team understood and respected Nepali culture **rarely / never**.
4. I'm not sure.

18. How much did COVID-19 impact your **personal / individual** ability to complete work with this project?

1. Not at all
2. A little
3. Somewhat
4. A lot
5. I'm not sure

19. How much did COVID-19 impact **the team’s** ability to complete work with this project?

1. Not at all
2. A little
3. Somewhat
4. A lot
5. I'm not sure

20. If you were asked to be part of another global research team, what would you say?

1. Yes, definitely.
2. Maybe, I'm not sure.
3. No, definitely not.

**Part 4. (6 questions)** 
Almost done!  This **final** set of questions will allow you the opportunity to provide more in-depth information about your experiences during this research project if you wish. As a reminder, there are no right or wrong answers! All answers are **anonymous** and will in **no way** be linked to you.

1. What do you feel is the **most important thing** you learned working on this project?

2. Is there anything you **wanted** **to learn** during this project, but **did not** get the chance to learn?

3. What did you like **best** about participating in this project?

4.What did you like **least** about participating in this project?

5. What activity did you find **most meaningful or helpful?**

6. Is there anything else you would like to share about your experience participating in this project?

Thank you so much for answering these questions!  

We are also interested in conducting individual interviews to learn more about your experience. 

If you are willing to be interviewed, please click below and you will be redirected to provide your contact information. (Please note: your contact information will **not** be connected to the answers you provided for this survey.)

 **Would you be willing to participate in a follow-up interview?**

1. Yes
2. No
