# Supplementary material for: Co-constructing collaboration: An evidence-based approach to advance and evaluate equitable global public health research partnerships
Source: PLOS Glob Public Health. 2023 Oct 23;3(10):e0002481. doi: 10.1371/journal.pgph.0002481 (PMC10593218; doi:10.1371/journal.pgph.0002481)
Supplement: S2 Text — (DOCX) [file pgph.0002481.s002.docx]

**S2 Text**

Semi-Structured Interview Guide

1. What motivated you to work on this research project?
2. How would you describe your involvement with the project?
3. What do you see as some of the benefits of research partnerships between lower/middle income countries and higher income countries (like the relationship with Nepal/U.S.)?

***Prompt****:  For individuals? For institutions? For the Nepal side? For the U.S. side?*

1. What do you think makes a good global research partnership?
2. What are some things that may make things difficult for a global research partnership?
3. (Unless specifically answered in 4) What is needed to make global partnerships equal and beneficial for both groups/countries?

***Prompt:*** *Do you feel this project had that? Can you elaborate on your experience?*

1. What do you think has been – or will be - the biggest, long-term impact of the project?

***Prompt:*** *Do you think this work will continue? Why or why not?*

1. How did COVID impact the project?

***Prompt****:  For individuals? For institutions? For the Nepal side? For the U.S. side?*

1. Looking back, if there is one thing you could change about this project what would it be?
2. What did you find most frustrating or stressful about working on this project?
3. What did you find most rewarding or enjoyable about working on this project?
4. Is there anything you’d like to add about your experience?
